# Identifying COVID-19 clusters in Tennessee long-term care facilities based on weekly staff vaccination rates

**DOI:** 10.1017/ash.2023.294

**Published:** 2023-09-29

**Authors:** Marissa Turner, Ashley Gambrell, Erin Hitchingham, Simone Godwin

## Abstract

**Background:** In September 2021, the CMS mandated that long-term care facility (LTCF) healthcare workers be vaccinated for COVID-19 unless medically or religiously exempt. Vaccinating healthcare workers reduces transmission of COVID-19 among patients and workers, reducing the risk of illness among residents and patients. We examined the relationship between COVID-19 clusters and staff vaccination rates in Tennessee LTCFs. **Methods:** COVID-19 cluster data were collected using REDCap from January 3, 2021, to September 25, 2022, and LTCF vaccination rates were collected from the NHSN. Clusters were identified in facilities with 2 or more cases. The staff vaccination rate 2 weeks prior to the cluster was used, accounting for the lag time between vaccination dose and reaching full immunity. We selected 75% as the critical immunization threshold. The facility case rate was calculated per 100 beds. A test was performed to determine whether reaching the critical vaccination threshold was associated with cluster occurrence. The relationship between vaccination rate and case number was tested using Pearson correlation. Statistical analyses were conducted using SAS version 9.4 software. **Results:** The average staff vaccination rate when NHSN first required long-term care facilities to report rates rose from 47% in June 2021 to 83% in September 2022. In total, 806 clusters were identified with 20,868 combined weeks from all facilities being reported after merging facilities’ weekly vaccine percentage rates with cluster data. Most weeks from all facilities did not identify a cluster (n = 20,064, 96.15%) and did not meet the critical immunization threshold (n = 11,050, 52.95%). The association between a cluster occurring and a facility meeting the threshold was significant (χ^2^ = 5.41; df = 1; *P* 95% CI, .7327–.9740). The Pearson correlation coefficient between vaccination rate and case number was 0.05560 (*P* = .2894). **Conclusions:** There was a significant association between facilities not reaching the immunization threshold and presence of a COVID-19 cluster. The facility case rate was not correlated with staff vaccination rate; however, a limitation of this analysis was that resident vaccination was not tested. Another limitation was that medical and religious exemptions could not be differentiated. Healthcare staff should consider getting vaccinated, if able, to reduce the risk of COVID-19 and to keep staff and residents safe from COVID-19.

**Disclosures:** None

